# Area Increase and Budding in Giant Vesicles Triggered by Light: Behind the Scene

**DOI:** 10.1002/advs.201800432

**Published:** 2018-06-05

**Authors:** Vasil N. Georgiev, Andrea Grafmüller, David Bléger, Stefan Hecht, Sonja Kunstmann, Stefanie Barbirz, Reinhard Lipowsky, Rumiana Dimova

**Affiliations:** ^1^ Department of Theory and Bio‐Systems Max Planck Institute of Colloids and Interfaces Science Park Golm 14424 Potsdam Germany; ^2^ Department of Chemistry & IRIS Adlershof Humboldt‐Universität zu Berlin Brook‐Taylor‐Str. 2 12489 Berlin Germany; ^3^ Physikalische Biochemie Universität Potsdam Karl‐Liebknecht‐Str. 24‐25 14476 Potsdam Germany

**Keywords:** azobenzene, lipid membranes, molecular dynamics, photoswitch, vesicles

## Abstract

Biomembranes are constantly remodeled and in cells, these processes are controlled and modulated by an assortment of membrane proteins. Here, it is shown that such remodeling can also be induced by photoresponsive molecules. The morphological control of giant vesicles in the presence of a water‐soluble *ortho*‐tetrafluoroazobenzene photoswitch (F‐azo) is demonstrated and it is shown that the shape transformations are based on an increase in membrane area and generation of spontaneous curvature. The vesicles exhibit budding and the buds can be retracted by using light of a different wavelength. In the presence of F‐azo, the membrane area can increase by more than 5% as assessed from vesicle electrodeformation. To elucidate the underlying molecular mechanism and the partitioning of F‐azo in the membrane, molecular dynamics simulations are employed. Comparison with theoretically calculated shapes reveals that the budded shapes are governed by curvature elasticity, that the spontaneous curvature can be decomposed into a local and a nonlocal contribution, and that the local spontaneous curvature is about 1/(2.5 µm). The results show that exo‐ and endocytotic events can be controlled by light and that these photoinduced processes provide an attractive method to change membrane area and morphology.

## 1 Introduction

Almost all cellular processes occurring at the level of the membrane involve dynamic remodeling. A couple of examples of such processes include endo‐ and exocytosis as well as vesicle shedding and budding during vesicular transport.^1^ Different morphological changes of the membrane are observed in the course of these processes.^1^, ^2^ The mechanical properties of the bilayer and the regulating role of associated biomolecules such as proteins and carbohydrates are key to modulating these shapes.^3^ When exploring such cellular processes, giant unilamellar vesicles (GUVs)^4^ serve as a well‐established system for mimicking the plasma membrane. Their sizes (in the range of 1–100 µm) make GUVs amenable to direct observation of the membrane behavior under a microscope in real time. The response of GUVs when exposed to various external stimuli such as temperature and osmotic stress,^5^ magnetic^6^ and electric fields,^7^ chemical reactions or pH gradients,^8^ polymers,^9^ and detergents^10^ (not to mention the numerous studies with peptides and proteins) has already been investigated. Experiments with GUVs have thus unequivocally demonstrated the utility of this system for unveiling the material properties of the lipid bilayer.

Molecules, which are able to reversibly interconvert between two different forms upon illumination, are another intriguing tool for reshaping the membrane. The use of these photoswitchable molecules thereby offers an approach for direct conversion of light into mechanical energy.^11^ Moreover, light‐responsive liposomes appear to be an attractive setup for a drug delivery system. For the latter application, one typically employs liposomes encapsulating a sufficient amount of therapeutic agents. These liposomes have the ability to carry the substance in aqueous fluids (bloodstream) and modulate the drug biodistribution.^12^ The light exposure of liposomes, made of photoswitchable lipids can indeed trigger a rapid release of the vesicle content.^13^

Azobenzene derivatives have been the most‐widely employed class of photoswitches for the photocontrol of biomolecules.^14^ In the past few years, the use of azobenzene derivatives for photocontrol of peptides,^15^ ion channels,^16^ nucleic acids, and oligonucleotides^17^ has been reported. Azobenzene‐modified lipids have been employed to study the membrane properties in model systems such as small unilamellar vesicles (SUVs) and large unilamellar vesicles (LUVs). Increase in the permeability of SUVs and LUVs^18^ as well as photocontrol of vesicular adhesion^19^ have been achieved.

Since GUVs are a convenient tool for directly exploring the response of the phospholipid membranes at the scale of a cell, a number of studies combining GUVs and light as an external stimulus have been also performed.^20^ Typical azobenzene derivatives undergo *trans* → *cis* photoisomerization induced by UV light. When exposed to UV light, multicomponent GUVs in the presence of azoTAB, a photosensitive cationic azobenzene surfactant, were observed to burst.[[qv: 20a]] This behavior was enhanced for membranes in the gel or liquid ordered phases compared to membranes in the liquid disordered phase. It was suggested that the latter membranes can reorganize fast and thus accommodate changes in the azoTAB configuration. Changes in the vesicle morphology using another type of azobenzene derivative with lipid tails, KAON12, causing reversible *exo*‐ and *endo*‐budding transitions upon irradiation with UV and visible light have been demonstrated in vesicles doped with KAON12.[[qv: 20b,c]] The effects were assigned to an increase in the membrane surface area upon photoisomerization. However, vesicles with similar initial shape did not always exhibit the same morphological transformations, which might suggest that the area increase is not the only parameter involved in this process. Vesicles made entirely from a phosphatidylcholine with one tail containing azobenzene (azo‐PC) were shown to undergo controlled morphological changes and deformability.[[qv: 20d]]

**Figure 1:**
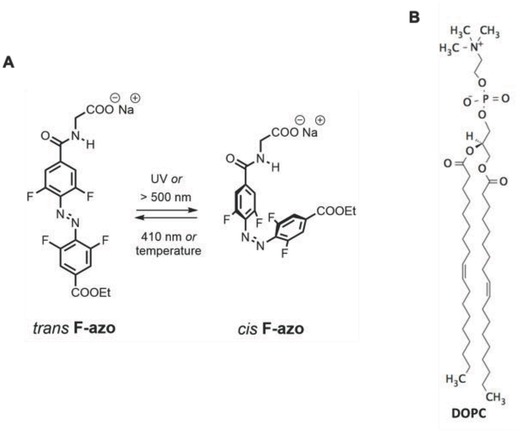
Molecular structures of A) F‐azo and B) DOPC. The *trans‐cis* photoisomerization of F‐azo takes place in the ranges of 300–375 nm or above 500 nm, while the *cis*–*trans* configurational change occurs in the range of 410–440 nm.

For all of the observed light‐triggered morphological changes in GUVs reported so far, only photoswitches that strongly intercalate in or even form the membrane have been employed. These are molecules, which are either practically insoluble in water, or have a surfactant‐like nature, and are thus more difficult to administer. Here, we investigate the possible mechanisms for lipid morphological transitions caused by light in the presence of a novel *ortho*‐tetrafluoroazobenzene derivative (F‐azo, see **Figure** **1**A), which is water‐soluble and mildly interacts with the membrane. Furthermore, contrary to parent azobenzene, F‐azo can undergo isomerization under visible light only,^21^ i.e., green/cyan for the *trans* → *cis* isomerization and blue/violet for the reverse process. Thus, the large structural changes can be achieved without the need of applying UV light, which is damaging to cells.^22^ Note that the necessity of UV light typically limits the practical use of azobenzenes in life and material science applications.^23^ In addition and contrary to the typical azobenzene surfactants carrying cationic ammonium groups, F‐azo is anionic with a carboxylate residue. This feature is expected to lower the cytotoxicity of the photoswitch. The cationic nature of azobenzene derivatives can mediate strong adhesion of biological samples to glass (e.g., microscope slides) leading to membrane rupture during in vitro studies, presumably affecting their interpretation. This problem can be circumvented by the use of F‐azo. Finally, F‐azo derivatives exhibit high thermal stability. Their *cis*‐isomer thermally converts to the more *trans*‐isomer very slowly (half‐life of 22 h at 60 °C in acetonitrile solution).^24^ The combination of these unique features of F‐azo opens the gate toward bulk applications, such as optomechanics of soft organic materials,^25^ the lipid membrane being just a first example.

In this work, we investigated the photoresponse of membranes exposed to F‐azo present in solution at submillimolar concentrations. Using a comprehensive set of experimental methods combined with molecular dynamics (MD) simulations and a theoretical description, we explored the possible mechanisms of photoinduced morphological changes in GUVs.

## 2 Results and Discussion

We used F‐azo molecules as photoswitches and investigated the influence of their photoisomerization on GUVs made of dioleoylphosphatidylcholine (DOPC). F‐azo is able to isomerize under visible light: *trans* → *cis* isomerization can be achieved with cyan (488 nm) and *cis* → *trans* with violet (420 nm) light, see Figure S1, Supporting information.^21^ The *trans‐*isomer is nearly planar, while the *cis*‐isomer has a distorted azo core with twisted phenyl rings attached (see Figure 1A). Presumably, because of the weak intensity of the light sources in the cyan range (see Figure S3, Supporting information) on the one hand, and the weak absorbance of F‐azo in this wavelength region (see Figure S1, Supporting information) on the other hand, the vesicles were not observed to respond to cyan irradiation. The use of a more powerful green light‐emitting diode (LED) did not induce responses of the GUVs either. We thus focused on the use of UV light (365 nm) instead to induce the *trans* → *cis* photoisomerization within the GUVs, while blue light (470 nm) was used to trigger the reverse *cis* → *trans* photoisomerization.

### 2.1 Vesicle Response to F‐azo Isomerization

GUVs prepared in 100 × 10^−3^ m solution of sucrose were mixed (volume ratio 1:1) with a solution of glucose and F‐azo to reach a final F‐azo concentration of 0.25 × 10^−3^ m. The molarity of the glucose/F‐azo solution (≈105 × 10^−3^ m) was set slightly higher than the one of the sucrose solution in order to deflate the GUVs, release their initial tension and render them quasi‐spherical, and with visible fluctuations. After 2 h of incubation with the F‐azo molecules, the GUVs were alternatively irradiated with UV (365 nm) and blue (470 nm) light and their behavior recorded.

**Figure 2:**
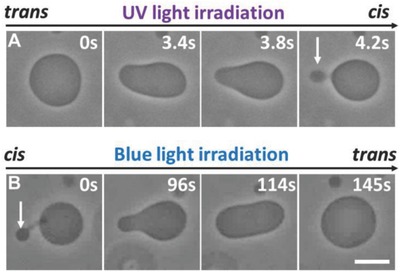
Dynamic response of one quasi‐spherical vesicle to photoisomerization of F‐azo at bulk concentration of 0.25 × 10^−3^ m. A) The vesicle undergoes outward budding under UV light irradiation (365 nm). The arrow points to the expelled bud. B) Shape transition of the same vesicle when exposed to blue light (470 nm). The bud is readsorbed and the vesicle attains its initial quasi‐spherical shape. The time after starting the irradiation is indicated on each snapshot. The scale bar represents 10 µm.

Upon exposure to UV light, all quasi‐spherical vesicles exhibiting visible fluctuations underwent morphological transitions in response to the photoisomerization of F‐azo (see **Figure** **2**A; Movie S1, Supporting Information). In comparison, no visible morphological changes were detected for vesicles, which appeared initially tense (spherical and without visible fluctuations). Under UV light, when the F‐azo molecules change their configuration from *trans* to *cis*, quasi‐spherical vesicles adopt a prolate shape followed by the expelling of a small bud (out‐bud, see Figure 2A). The latter remains connected to the vesicle via a thin neck. The process is reversible and the out‐bud is readsorbed into the vesicle body under blue light irradiation (Figure 2B), yet at a slower rate. Note that the exchange of the filters for UV and blue light takes ≈15 s and constant monitoring between changes in the irradiation was not possible. Under UV irradiation, vesicles with diameter of 10–20 µm undergo budding in the first 4–15 s. The vesicles remain prolate (do not expel buds) if the UV light is switched off after only a few seconds of irradiation, and after a few minutes restore their quasi‐spherical shape. The bud readsorption under blue light takes typically ≈2 min to complete. Note that this process occurs also in the absence of blue light, but takes longer time (≈4 min). The morphological cycles can be repeated without any significant differences (during different cycles, the buds may form on different parts of the same vesicle). Ten cycles of this reversible morphological change could be observed (data not shown). The reversibility in morphology suggests that no effects associated with photooxidation (such as those observed, e.g., in ref. 26) are present.

We examined not only vesicles, which were dispersed in F‐azo solutions after formation as discussed above, but also vesicles grown in the presence of F‐azo (0.25 × 10^−3^ m). The same response was observed suggesting that in the experiments with vesicle incubation in F‐azo solutions, the system is equilibrated and the F‐azo molecules are equally distributed between the internal (enclosed into the vesicles) solution and the external one and (or) between the two leaflets of the membrane.

In the absence of F‐azo, no changes were detected when the vesicles were exposed to UV light for 1 min (see Figure S4, Supporting Information). In addition, the UV irradiation was not found to change the temperature in the observation chamber. Thus, UV light irradiation alone cannot cause the observed shape transformations in our system.

### 2.2 F‐azo Partitioning in the Membrane

**Figure 3:**
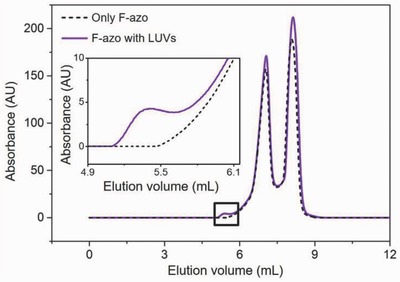
Elution profiles of F‐azo molecules in the absence of LUVs (black dashed curve) and after incubation with LUVs for 2 h (purple solid curve) measured by absorbance at 320 nm. 50 µL of the sample were loaded in the column with volume of 24 mL. The elution peak at ≈5.4 mL (magnified in the inset) corresponds to F‐azo inserted in the membrane. The F‐azo and lipid concentrations for all experiments were 0.25 × 10^−3^ and 0.1 × 10^−3^ m, respectively.

Size‐exclusion chromatography (SEC) as well as dynamic light scattering (DLS) of F‐azo solutions at our working concentrations showed the presence of two populations of aggregates with a broad size distribution (**Figure** **3**, see also Figure S5, Supporting Information). The SEC traces show that F‐azo eluted as two peaks with maxima at ≈7 and ≈8.1 mL as detected via UV‐absorbance at 320 nm. LUVs without F‐azo eluted earlier, at ≈5.5 mL (see Figure S6, Supporting Information) as monitored via fluorescence. Only when vesicles were mixed with F‐azo we could detect an additional UV‐peak, which corresponds to a signal from F‐azo molecules incorporated in the lipid bilayer of the LUVs. Its low intensity is in line with the large excess of free (i.e., not membrane‐incorporated) F‐azo as compared to the lipid concentration. From the peak areas, we calculated the concentration of F‐azo incorporated in LUVs to be 1.85 × 10^−6^ ± 0.13 × 10^−6^ m, corresponding to 0.74% ± 0.05% of the total F‐azo present in the sample. This implies on average an insertion of one F‐azo molecule for every 54 ± 4 lipid molecules.

### 2.3 Partitioning and Flip‐Flop Free Energy of F‐azo Obtained by MD Simulations

**Figure 4:**
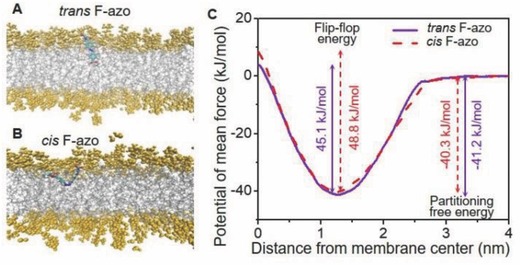
MD simulations of one F‐azo molecule in a DOPC bilayer composed of 256 lipids. A,B) Orientation of *trans* and *cis* F‐azo in the membrane. DOPC head groups are shown in yellow, tails in gray. The bonded structure of F‐azo is color‐coded by atom (red: O, cyan: C, white: H, blue: N, rose: F). C) PMF for *trans* and *cis* F‐azo.

To assess the distribution of F‐azo between the solution and the membrane, and between the two leaflets of the membrane, we performed MD simulations (see Experimental Section). Snapshots of the DOPC bilayer with the two different isomers are shown in **Figure** **4**A,B. The amphiphilic nature of F‐azo results in positioning the molecule at the head group—tails interface. The *trans* F‐azo isomer tends to penetrate deeper into the membrane than the molecule in *cis* configuration, and to orient along the bilayer normal, whereas the *cis* isomer aligns at the head–tail interface (see Figure 4A,B).

The profiles of the potential of mean force (PMF) to displace the F‐azo molecule from the water phase to the center of the bilayer at *z* = 0 nm are displayed in Figure 4C. The partitioning free energies, Δ*G*_min_ corresponding to the depths of the potential minima, are similar Δ*G*_min_ ≈ −40 ± 5 kJ mol^−1^ for both the *trans‐* and *cis‐*isomer. This value suggests that the molecules insert into the membrane regardless of their configuration. These partitioning free energies are lower than those typical for lipids and cholesterol (70–80 kJ mol^−1^)^27^ suggesting that the molecular cohesion of F‐azo to the lipids in the bilayer is poorer and that the photoswitch partitions more weakly than lipids do. However, no desorption from the bilayer resulting from the configurational change of F‐azo is expected.

The profile of the potential of mean force also suggests that the energy barrier for flip‐flop of F‐azo is between 45 and 49 kJ mol^−1^ (see Figure 4C). This value is higher than the one of cholesterol ≈24 kJ mol^−1^,^27^ but it is lower than that of lipids ≈78 kJ mol^−1^.^28^ The typical timescale for cholesterol flip‐flop in polyunsaturated bilayers is less than 1 s, while the flip‐flop time of fluorescent lipid analog in a membrane made of DOPC (as in our model system) is ≈1 h.^27^, ^29^ Since the obtained energy barrier for flip‐flop of F‐azo has an intermediate value, we expect that the flip‐flop rate of the molecules in our system is in the time range of 1 s–60 min. Using the dynamic constants reported in ref. 27 for cholesterol in a dipalmitoylphosphatidylocholine bilayer would lead to a timescale of ≈1–10 s for F‐azo flip‐flop. Thus, we conclude that within the incubation time of the GUVs with F‐azo (1–2 h), the photoswitches are distributed in both membrane leaflets. This is consistent with the finding that the morphological changes observed with incubated vesicles (F‐azo added postpreparation) are identical to the ones with vesicles formed in the presence of F‐azo.

### 2.4 Area Change Caused by F‐azo Isomerization

From the obtained morphological changes of quasi‐spherical vesicles, we speculated that the area of the vesicle increases during the photoisomerization of F‐azo in the membrane. To assess this area increase, we employed vesicle electrodeformation, see the Experimental Section. The GUVs were formed in the presence of salt and diluted with salt‐free solution to establish higher conductivity inside. This condition ensures prolate deformation in vesicles exposed to AC field.[[qv: 7a]] The GUVs were deflated by diluting them in a solution composed of glucose (≈105 × 10^−3^ m) and the desired concentration of F‐azo. After an incubation period of 2 h, the vesicles were exposed to electric field which deforms them and pulls out the membrane fluctuations. The total vesicle area can be calculated from the ellipsoidal vesicle shape. Subsequently, the vesicles were exposed to UV light (with the AC field still on) and the change in the vesicle area recorded.

Because of the sugar asymmetry (sucrose inside and glucose outside), after preparation, the vesicles appear dark on a lighter background when observed under phase contrast (Figure 2). Neither the incubation with F‐azo, nor the vesicle electrodeformation, nor the irradiation caused loss of contrast which is an indication that the membrane remains intact throughout the experiments.

**Figure 5:**
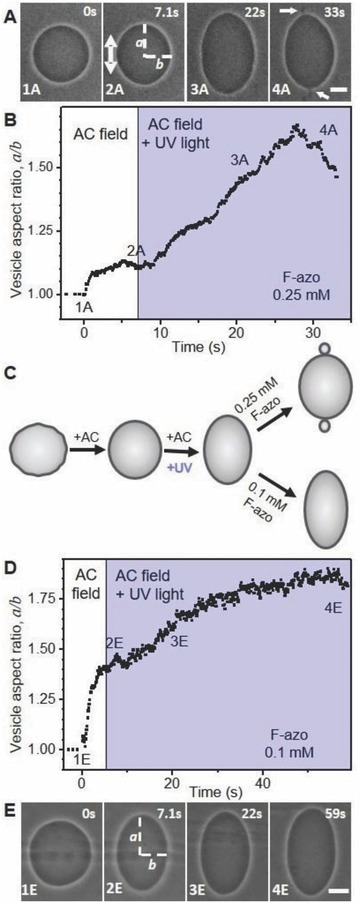
Deformation and budding of vesicles caused by F‐azo isomerization in the membrane at two different F‐azo concentrations: A,B) 0.25 × 10^−3^ m and D,E) 0.1 × 10^−3^ m. The graphs in (B) and (D) show the vesicles degree of deformation (*a*/*b*) over time. Schematic illustration of the vesicle shape changes is shown in (C). The snapshots in panels (A) and (E) correspond to the time frames indicated on the upper left corner. (1A and 1E) Vesicle in the absence of field, (2‐4 A and E) the vesicle is exposed to an AC field (10 kV m^−1^ and 1 MHz). The direction of the field is indicated in snapshot 2A. The UV irradiation (violet regions in B and D) starts after 7.1 s (2A and 2E). At the higher F‐azo concentration, the vesicle expels two buds (see arrows in the snapshot in Figure 4A with the scale bars corresponding to 10 µm).

The degree of deformation of the vesicles in AC field and in the absence of UV irradiation (compare the images and data in **Figure** **5**A,E vs Figure 5B,D) can vary and this is not related to the overall concentration of F‐azo but depends on the initial excess area available for deformation (or volume‐to‐area ratio), which is different for every vesicle in the sample. This excess area is quantitatively represented by the reduced vesicle volume (i.e., the ratio between the vesicle volume and the volume of a sphere of equal area) defined as

(1) $$\nu \;=\;\frac{6\sqrt{\pi \;}V}{A^{3/2}}$$

where *A* is the total membrane area and *V* is the vesicle volume, which can be accessed from the ellipsoidal shape of the vesicle exposed to AC field. Between 5 and 7 s are typically sufficient for a vesicle (of typical radius ≈10–20 µm) in the absence of F‐azo to attain its maximal deformation (see Figure S7, Supporting Information). As calculated from the maximal achieved deformation in AC field for the vesicle in Figure 5A (at 7.1 s after switching the field on), the reduced volume is ν ≈ 0.996, while the reduced volume of the vesicle in Figure 5E is ≈0.967 (smaller reduced volume correspond to more excess area). The electrodeformation experiments showed that the UV irradiation of vesicles in the presence of F‐azo leads to an increase in the membrane area as a result of the *trans*‐to‐*cis* F‐azo isomerization. Figure 5 shows the relative area increase in GUVs for two different F‐azo concentrations. Since our measurements on F‐azo partitioning in the membrane and vesicle morphological changes were performed with 0.25 × 10^−3^ m F‐azo, we first explored the vesicle area change at this concentration, see Figure 5A,B. The UV irradiation is initiated at 7.2 s. After around 22 s, the vesicles are typically no longer elliptical. They adopt lemon‐like shapes and start budding in the area of the poles facing the electrodes (see last snapshots in Figure 5A). The budding process is reversible in the presence of an AC field when the vesicle is irradiated with blue light (see Figure S8, Supporting Information). The exact area can be deduced from measuring the two semiaxes, *a* and *b* of the vesicle when it is still an ellipse. For this vesicle, the relative area increase before budding (after 22 s of irradiation) was 4.6%, see Equation (4). During and after budding, the vesicle area cannot be deduced correctly from the phase‐contrast microscopy images because of the small size of the buds. The average relative area increase ≈22 s after the irradiation (i.e., shortly before budding) measured on 30 different vesicles from different preparation batches is 4.3% with standard error of mean ± 0.4%. This is obviously a lower‐limit estimate of the maximal area increase.

The same experiment was performed also at a lower concentration of F‐azo (0.1 × 10^−3^ m). At this concentration, most of the vesicles did not expel buds and the GUVs remained elliptical attaining their maximum deformation upon UV irradiation. We used the data to deduce the total area change in the vesicles caused from F‐azo isomerization. Between 55 and 60 s were sufficient for the vesicles to reach their maximal deformation. The maximal area increase for the example in Figure 5D,E, is 5.1%. For 30 different vesicles from different preparation batches, we found that the average maximal area increase is 4.8% with standard error of mean ± 0.5%.

To be able to compare the results for the two concentrations, we also measured the relative area change ≈22 s after the irradiation (i.e., before budding is observed for the samples with 0.25 × 10^−3^ m F‐azo). Under these conditions the average relative area increase in the presence of 0.1 × 10^−3^ m F‐azo is 2.4% ± 0.3%. This value is lower than the one measured in the presence of 0.25 × 10^−3^ m F‐azo (4.3%) because of the lower F‐azo fraction in the membrane.

MD simulations can also be used to predict the area increase when the molecules undergo *trans*‐to‐*cis* photoisomerization. A series of simulations of DOPC bilayer patches containing between 2 and 10 F‐azo molecules symmetrically distributed in both leaflets (and corresponding to the range between 128 and 25 lipids per F‐azo molecule) show a maximum area increase of Δ*A* ≈ 1.6% when 10 F‐azo molecules were added. Although the effect is smaller than the experimentally measured increase, it illustrates how the configurational change of the molecules can lead to the observed increase in the vesicle area. As the area increase in the experiments is seen to continue after seconds, the different magnitude of the observed effect may be related to the much smaller length and time scales in the simulated system.

The configurations observed in the MD simulations suggest that two factors contribute to the measured area increase induced by the *trans*‐to‐*cis* isomerization: First, as the *trans* isomer orients itself approximately parallel to the bilayer normal and the two phenyl rings present a relatively flat surface, it has an ordering effect on the lipid tails surrounding it, similar to cholesterol. The *cis* isomer on the other hand lies parallel to the head–tail interface, and has no measurable effect on the chain order. In addition, the *trans* molecules show a tendency to aggregate inside the bilayer, with the phenyl rings stacked next to each other, further reducing the area per molecule, whereas no such clustering is observed for the *cis* molecules (see also Figure S9, Supporting Information). The clustering of molecules in the bilayer is a slow process, which is not equilibrated on the timescale of the simulations, and therefore likely to be a factor contributing to the observed difference in the magnitude of the area increase connected with the *trans*‐to‐*cis* transition.

### 2.5 Spontaneous Curvature and Budding Condition

The shape of a vesicle depends primarily on the volume‐to‐area ratio, as defined by the reduced volume ν (Equation (1)) and on the dimensionless spontaneous curvature^30^

(2) $$\bar{m}\equiv m\;R_{ve}=\;m\sqrt{A/\left(4\pi \right)}$$

which represents the product of the spontaneous curvature *m* and the vesicle size *R_ve_*.

**Figure 6:**
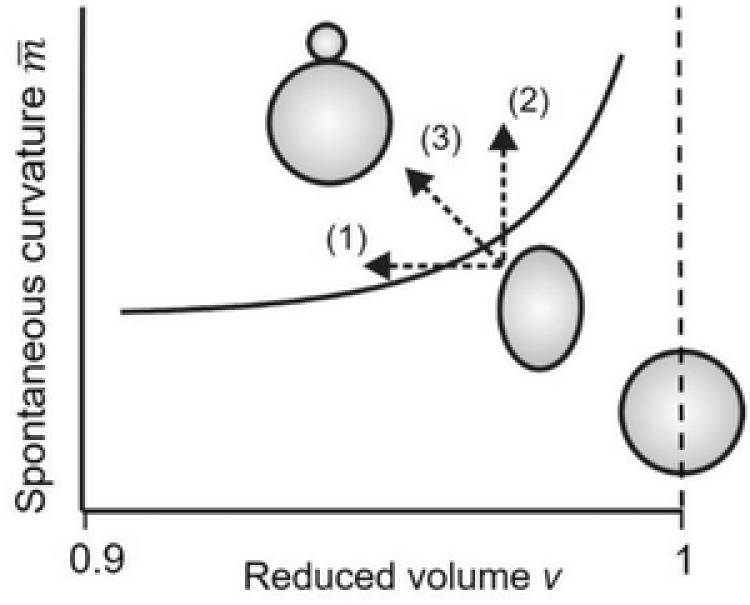
Schematic morphology diagram as a function of reduced volume ν and dimensionless spontaneous curvature $\bar{m}$. The solid curve represents the line of limit shapes at which the membrane necks of the bud close. When the F‐azo‐doped membrane is exposed to UV irradiation, the prolate‐shaped vesicle may follow three different pathways as indicated by the dashed arrows: (1) An increase of the vesicle area only, leading to a decrease in the reduced volume ν, (2) An increase in the spontaneous curvature for fixed reduced volume, and (3) An increase in membrane area which leads to a decrease of ν and an increase of the dimensionless spontaneous curvature $\bar{m}$.

A partial morphology diagram of the vesicle shapes as a function of these two parameters^31^ is shown in **Figure** **6**. For ν = 1 the vesicle is a sphere. For ν < 1, the excess area of the vesicle can lead to shape transformations. The solid curve schematically represents the boundary between buds with open and with closed membrane necks.

Possible pathways, which the vesicles can take upon F‐azo isomerization are represented by the trajectories in the morphological diagram (Figure 6) as follows: 1) increase in the membrane area only (decrease in the reduced volume, ν < 1) at a positive and constant spontaneous curvature, 2) only the membrane spontaneous curvature increases at constant reduced volume, whereby ν < 1, and 3) both the spontaneous curvature and vesicle area increase (ν decreases).

During our experiments with photo irradiation in the presence of F‐azo, the GUVs did not lose their optical contrast (resulting from the difference in the refractive index between the internal and the external solutions of the vesicles), which strongly indicates that the membrane remains intact and that the vesicle volume does not change. Image analysis of the vesicles before and after budding confirms that the volume remains constant within the detection error. Therefore an increase in the vesicle area, as detected from vesicle electrodeformation experiments (Figure 5), is the only source for changing the reduced volume. Since the reduced volume decreases (increasing area), we can exclude pathway (2) in Figure 6. The vesicles undergo outward budding when exposed to UV light, which implies a positive spontaneous curvature. The sizes of the buds were in the micrometer range indicating small positive values of the spontaneous curvature.

**Figure 7:**
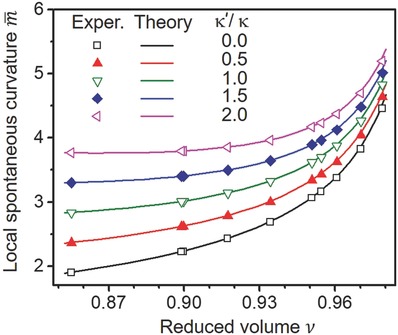
Local spontaneous curvature $\bar{m}$ as defined in Equation (2) as a function of the reduced volume *v* for different values of the rigidity ratio *κ ′/κ*. For a given value of this ratio, all experimental data collapse onto the corresponding theoretical curve.

Budding can arise from an asymmetry in the bilayer leaflet compositions and/or different solution composition on the two sides of the membrane. We first consider solution asymmetry. Indeed, trans‐bilayer sugar asymmetry leading to adsorption or depletion layers (sucrose inside and glucose outside as in the above experiments) can generate spontaneous curvature of the order of the one we observe (see **Figure** **7**).^32^ However, this is not the only source of spontaneous curvature here, because when prepared and irradiated in symmetric glucose conditions, vesicles with F‐azo bud outward as well (see Section S9, Supporting Information). Asymmetry in the solutions, especially in terms of the aggregates detected with size exclusion chromatography (see Figure 3) and dynamic light scattering (see Section S4, Supporting Information) is a more probable source of the spontaneous curvature induced by depletion layers. Indeed, we could reverse the budding direction in vesicles grown in symmetric sugar conditions in the presence of F‐azo, after diluting them to reduce the external concentration of F‐azo (see Section S9, Supporting Information). Further below, we consider possible contributions from leaflet area asymmetry.

The equilibrium vesicle shapes can be obtained by minimization of the bending energy as described by the spontaneous curvature model^30^, ^33^ or the area‐difference‐elasticity model^31^, ^34^ see Section S10, Supporting Information.

The shapes that minimize the energy functionals of both models require the introduction of an effective spontaneous curvature *m*_eff_ = *m* + *m*_nlo_ which represents the sum of the local spontaneous curvature *m* and the nonlocal spontaneous curvature *m*_nlo_.^31^ The latter contribution is proportional to the difference between the integrated mean curvature of the initial shape and of the budded shape.

In the experiments, the budded shapes are well described by a small spherical bud and a larger spherical mother vesicle. The neck connecting the two spheres is then governed by the neck condition (see Section S10 of the Supporting Information for derivation)

(3) $$2m\;=\frac{1}{R_{\alpha }}\;+\;\frac{1}{R_{\beta }}-\;2\pi \frac{\kappa^{}\prime }{\kappa }\frac{\sqrt{R_{\alpha }^{2}+R_{\beta }^{2}}\;-\;R_{\alpha }\;-\;R_{\beta }\;}{R_{\alpha }^{2}\;+\;R_{\beta }^{2}}$$

where *R*_α_ and *R*_β_ are the radii of the mother vesicle and the bud, and, κ ′ and κ are the nonlocal and the local bending rigidity, respectively. The term of Equation (3) that is proportional to the rigidity ratio κ ′/κ represents the nonlocal spontaneous curvature which favors a certain area difference between the two leaflets if the membrane molecules cannot undergo flip‐flops between these two leaflets. This preferred area difference is provided by the area difference of the initial vesicle shape.

We measured *R*_α_ and *R*_β_ for ten GUVs, which underwent morphological transition (from a spherical to an out‐budded shape) upon UV irradiation in the presence of F‐azo. Then, following Equation (3), we calculated the local spontaneous curvature for different values of the bending rigidity ratio κ ′/κ. The experimentally obtained *m* values for these vesicles are plotted in Figure 7 as a function of their reduced volume (Equation (1)). We also calculated the lines of budded shapes within the (ν, $\bar{m}$)‐plane while varying the term κ ′/κ between zero and two, see Figure 7.

For a given value of κ ′/κ, all experimental data for the budded vesicles collapse onto the corresponding theoretical curve that follows from Equation (3). As a consequence, all budded vesicles were characterized by the same value of the rigidity ratio κ ′/κ. In the Supporting Information (Section S10), we display the histograms for the nonlocal and the local contributions to the spontaneous curvature using the rigidity ratios κ ′/κ = 0 and κ ′/κ = 1. Inspection of these histograms reveals that the non‐local spontaneous curvature is negative while the local spontaneous curvature has the value *m* ≅ 1/(2.8 ± 1.0 μm) for κ ′/κ = 0 and the value *m* ≅ 1/(2.4 ± 0.8 μm) for κ ′/κ = 1 (see Figure S11).

## 3 Conclusion

In this work, using GUVs as a plasma membrane‐model system and employing light as an external stimulus in combination with photoswitchable molecules as transmitters of the light energy, we have investigated the dynamics of a process essential for intra/intercellular trafficking. We have implemented an anionic azobenzene derivative molecule (F‐azo) as the photoswitch. Of particular interest is the mechanism of the observed light‐induced morphological changes in GUVs, caused by *trans* → *cis* photoisomerization of the F‐azo molecules. Employing both theoretical and experimental approaches, we could show that the F‐azo molecules induce a significant (visible) increase in the membrane area and a positive membrane spontaneous curvature, which result in the observed outward budding events. We also developed a theoretical description of the observed budding events. Experimental data for the bud size was matched to a theoretically predicted dependence of the spontaneous curvature on the reduced volume. This provided a way of assessing the contribution of the nonlocal spontaneous curvature resulting from the asymmetry in the area of the leaflets.

Our efforts to establish the morphological changes in the vesicles solely using visible light were only partially successful. Probably the most advantageous feature of the photoswitches explored here is the fact that they are thermally reasonably stable, water‐soluble, and only mildly interact with the membrane, preventing vesicle bursting as reported earlier for cationic photoswitching surfactants.[[qv: 20a]] Remarkably, only minor fractions of F‐azo inserted in the lipid bilayer (a few mol%) are sufficient to trigger significant area increase in the membrane. The ability of F‐azo to isomerize in the visible spectrum could be a further step in the in vivo application of photoswitches. Increasing the molar extinction of such photoswitches in the green region and possibly shifting their absorbance more to the red,^21^ could possibly circumvent the nonresponsivity of the F‐azo‐doped vesicles in the >500 nm region. More studies in this direction are needed.

## 4 Experimental Section

*Vesicle Preparation*: GUVs were prepared from DOPC (Figure 1B) with the conventional method of electroformation[[qv: 4a]] while LUVs were prepared with extrusion; see Section S11, Supporting Information). The LUV size distribution was examined with DLS, see Section S11 in the Supporting Information. The F‐azo solutions at millimolar concentrations were observed to contain microscopic crystalline precipitates, which were filtered (see Figure S12 and Section S12, Supporting Information) not to obstruct imaging. Submicroscopic aggregates were still detected (with DLS) in the filtered F‐azo solutions (Figure S4, Supporting Information). The formation of microscopic crystalline aggregates could also be observed in filtered F‐azo solutions when subjected to UV irradiation for more than 2 min (Section S13, Supporting Information). The UV irradiation to less than 1 min was thus limited.

*Giant Vesicle Imaging and Irradiation*: The GUVs were observed under phase contrast mode of an Axio Observer D1 (Zeiss, Germany) microscope, equipped with a Ph2 20 × (NA 0.5) objective. Images were taken with an ORCA R2 CCD camera (Hamamatsu, Japan). The samples were irradiated using the HBO 100W mercury lamp of the microscope in epi‐illumination mode (the lamp spectrum is given in Section S2, Supporting Information). For UV, cyan and blue irradiation, the light from the mercury lamp passed through 365, 488/20, and 470/40 nm filter, respectively. The irradiation power of the HBO lamp was 61 mW cm^−2^ for the UV filter set (measured at 365 nm, see Figure S3, Supporting Information), ≈18 mW cm^−2^ at 488 nm, and 28 mW cm^−2^ for the blue filter (measured at 470 nm). Occasionally, more powerful cyan‐light sources were employed, including an in‐house build device with 505 nm LED source (≈27 mW cm^−2^). The power intensities were measured above the objective at the respective focal distance with LaserCheck power meter (Coherent, CA).

*Vesicle Electrodeformation*: Application of an AC field to GUVs was used to measure the relative area increase resulting from photoisomerization of the F‐azo molecules. The approach is based on the deformation of vesicles when exposed to the field^35^ and is similar to an approach reported earlier.^26^ GUVs grown in the presence of NaCl (0.5 × 10^−3^ m) were exposed to an AC field (10 kV m^−1^ and 1 MHz) applied in an electrofusion chamber (Eppendorf, Germany) with parallel cylindrical electrodes (92 µm radius), spaced at 500 µm. Under these conditions, the vesicles elongate and adopt a prolate shape with long axis parallel to the field direction.[[qv: 7d,35]] The experiment proceeds with first applying an AC field to a selected vesicle. The field deforms the vesicle into an ellipsoid, pulling out excess area stored in fluctuations. The total vesicle area, *A*, can be calculated from the vesicle shape. Then, the vesicle is exposed to UV light and the resulting relative area increase Δ$\bar{A}$ is calculated from

(4) $$\Delta \bar{A}=\;\frac{A_{\mathrm{UV}}\;-\;A}{A}$$

where *A*_UV_ is the area of the vesicle when exposed to UV light.

*Molecular Dynamics Simulations*: MD simulations were performed using parameters from the AMBER Lipid14 force field for DOPC[[qv: 7d,36]] and parameters for the F‐azo molecules from ref. 37 based on the general AMBER force field.^38^ Partial charges were derived for the *cis‐* and *trans*‐isomers separately using the R.E.D. tool scripts.^39^ Structure optimization of the molecule was performed with Gaussian at the HF/6‐31G* level of theory, and the final set of charges was obtained from an ensemble average of 50 structures generated from a 20 ns MD trajectory. The topologies for F‐azo and DOPC were converted using the glycam2gmx.pl script.^40^ The system was solvated with TIP3P water.^41^ All systems contained 256 DOPC and 16800 water molecules.

The PMF of the *z*‐coordinate of the F‐azo aromatic rings relative to the center of mass of the bilayer, were performed using umbrella sampling. Initial structures for 41 umbrella windows between *z* = 0 nm and *z* = 4 nm were generated for both the *cis* and *trans* configuration by pulling the molecule from its equilibrium position with a velocity of 0.211 nm ns^−1^ and a force constant of 200 kJ mol^−1^. Each window was equilibrated for 10 ns with a force constant of 200 kJ mol^−1^. Data were collected from 50 ns trajectories using a force constant of 1000 kJ mol^−1^. The PMF was constructed using the weighted histogram analysis method.

To obtain the area per molecule change upon isomerization, simulations with 2, 4, 6, 8, and 10 F‐azo molecules in the *cis* and in the *trans* configuration, distributed evenly between the leaflets were performed for 300 ns.

All simulations were performed in the NPT ensemble with GROMACS 4.6.4.^42^ Covalent bonds involving hydrogens were constrained using LINCS^43^ while water molecules were kept rigid with SETTLE,^44^ allowing the use of a 2 fs time step. Systems were equilibrated using the v‐rescale^45^ thermostat and Berendsen barostat.^46^

For data collection, the temperature and the pressure of the equilibrated systems were controlled with the Nose–Hoover thermostat^47^ and Parinello–Rahmann barostat^48^ with semi‐isotropic pressure coupling, respectively. Lennard‐Jones and short‐range electrostatic interactions were cut‐off at 1.0 nm, long range electrostatics were calculated using the particle mesh Ewald method.^49^

*Size‐Exclusion Chromatography*: SEC was performed on a Superdex 200 10/300 GL column (GE Healthcare, Freiburg, Germany) with a high‐performance liquid chromatography system (Shimadzu, Duisburg, Germany) equipped with a fluorescence detector and diode array UV‐detection. Before loading on the column, all samples were degassed and F‐azo molecules were centrifuged at 40 000 rpm for 30 min at 15 °C.

Prior to mixing with F‐azo, the extruded LUVs diameter was ≈140 nm as measured by DLS. Vesicles (0.1 × 10^−3^ m) were incubated for 2 h with F‐azo before being loaded on the column to reach an equilibrium of partitioning. As a mobile phase, 0.1 m sucrose/glucose solution was used. In each run, 50 µL F‐azo and lipid were applied at 0.25 × 10^−3^ and 0.1 × 10^−3^ m, respectively. Fluorescently labeled LUVs (containing 1 mol% DPPE‐Rh, see Section S11, Supporting Information) were detected with excitation at 550 nm and emission at 580 nm. Absorbance of F‐azo molecules was monitored at 320 nm.

## Conflict of Interest

The authors declare no conflict of interest.

## Supporting information

Supplementary

Supplementary
