# Supplementary material for: Area Increase and Budding in Giant Vesicles Triggered by Light: Behind the Scene
Source: Adv Sci (Weinh). 2018 Jun 5;5(8):1800432. doi: 10.1002/advs.201800432 (PMC6096984; doi:10.1002/advs.201800432)
Supplement: Supplementary file 1 — Supplementary [file ADVS-5-1800432-s002.pdf]

Copyright WILEY-VCH Verlag GmbH & Co. KGaA, 69469 Weinheim, Germany, 2018.

ADVANCED SCIENCE
Open Access

Supporting Information

for *Adv. Sci.,* DOI: 10.1002/advs.201800432

Area Increase and Budding in Giant Vesicles Triggered by Light: Behind the Scene

*Vasil N. Georgiev, Andrea Grafmüller, David Bléger, Stefan Hecht, Sonja Kunstmann, Stefanie Barbirz, Reinhard Lipowsky, and Rumiana Dimova**

Supporting information

Area Increase and Budding in Giant Vesicles Triggered by Light: Behind the Scene

Vasil N. Georgiev[†], Andrea Grafmüller[†], David Bléger[‡], Stefan Hecht[‡], Sonja Kunstmann[†,§], Stefanie Barbirz[§], Reinhard Lipowsky[†], Rumiana Dimova[†,*]

[†]Department of Theory and Bio-Systems, Max Planck Institute of Colloids and Interfaces, Science Park Golm, 14424 Potsdam, Germany

[††]Department of Chemistry & IRIS Adlershof, Humboldt-Universität zu Berlin, Brook-Taylor-Str. 2, 12489 Berlin, Germany

[§]Physikalische Biochemie, Universität Potsdam, Karl-Liebknecht-Str. 24-25, 14476 Potsdam, Germany

* Address for correspondence: dimova@mpikg.mpg.de

Table of Contents

S1. Synthesis and characterization of F-azo 2

S1.1. General information 2

S1.2. Photoisomerization of F-azo 2

S1.3. Synthesis 3

S2. Spectral distribution of light emitted by HBO 100 mercury lamp 4

S3. Effect of UV light (365 nm) on GUVs in the absence of F-azo. 5

S4. Size distribution of F-azo aggregates 5

S5. Elution profile of LUVs and F-azo molecules 5

S6. Electrodeformation of a GUV caused by AC field 6

S7. Reversible budding in the presence of an AC field. 6

S8. MD simulations to investigate the area increase produced by the F-azo molecules 8

S9. Effect of solution asymmetry on budding direction 8

S10. Spontaneous curvature of outward budding GUVs 9

S11. Materials and preparation of vesicles 10

S11.1. Materials 10

S11.2. Vesicle preparation 11

S11.3. Dynamic light scattering (DLS) of F-azo samples and LUVs 11

S12. F-azo aggregates 11

S13. Aggregation in filtered F-azo solutions under UV light irradiation 12

S14. Movie S1. Reversible morphological changes in a GUV 13

S15. References 13

S1. Synthesis and characterization of F-azo

S1.1. General information

Materials were purchased from commercial suppliers and used without further purification. Solvents were either used as received or dried employing an Innovative Technologies solvent purification system. Reactions were monitored by thin layer chromatography (TLC) carried out on silica gel plates (Merck 60F - 254) using UV light for visualization. Silica gel (Merck 60/VWR, particle size 0.040–0.063 mm) was used for column chromatography. NMR spectra were recorded on a Bruker 300 MHz spectrometer using residual protonated solvent signals as the internal standard (^{1}H-NMR: δ (CD_2Cl_2) = 5.32 ppm, δ (DMSO-d_6) = 2.50 ppm). Multiplicities are abbreviated as follows: s = singlet, d = doublet, t = triplet, q = quartet, m = multiplet. Ultraperformance liquid chromatography coupled to mass spectrometry detection (UPLC-MS) was performed with a Waters Alliance systems (gradient mixtures of acetonitrile/water) equipped with Acquity UPLC columns. The Waters systems consisted of a Waters Separations Module 2695, a Waters Diode Array detector 996, a LCT Premier XE mass spectrometer, and a Waters Mass Detector ZQ 2000.

S1.2. Photoisomerization of F-azo

UV/Vis absorption spectra were recorded using quartz cuvettes on a Cary 50 spectrophotometer equipped with a Peltier-thermostated cell holder (temperature accuracy ±0.1 K). The solvents used were of spectrophotometric grade. Irradiation experiments were performed using a LOT-Oriel 1000 W medium-pressure Xe/Hg lamp equipped with band-pass filters.

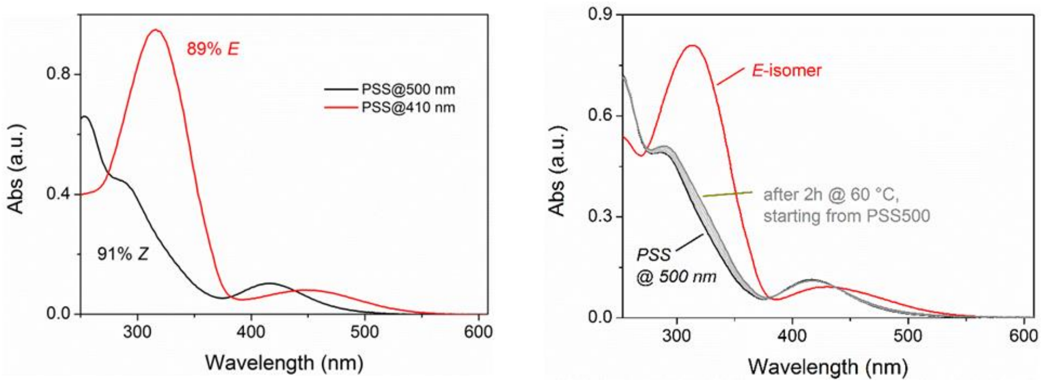

Figure S1. UV-Vis absorbance spectra of F-azo in water (ca. 4 x 10^{-5} M) at 25 °C. Left: spectra of the photostationary states (PSSs) with the compositions (in %) as determined by UPLC. Right: thermal $Z \rightarrow E$ isomerization at 60 °C.

S1.3. Synthesis

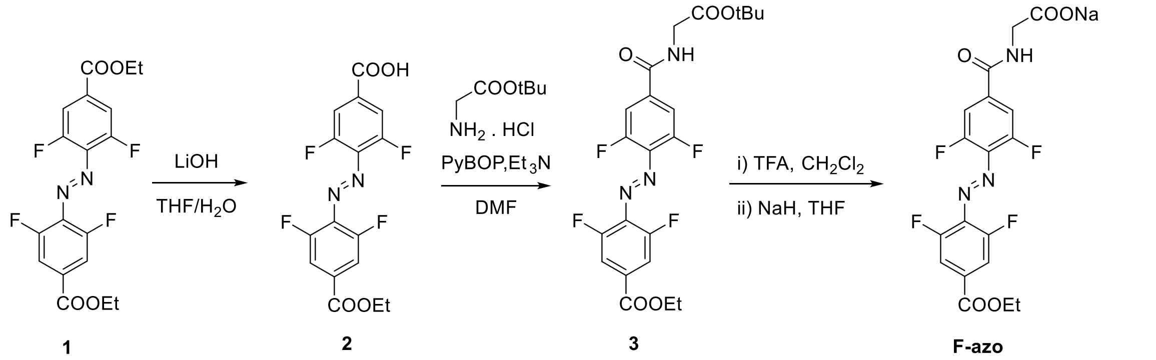

Scheme S1. Synthetic path to F-azo.

2: A solution of **1**[1] (1.37 mg, 3.44 mM) and LiOH (1.44 mg, 3.44 mM) in H_2O/THF 2:1 (36 mL) was heated at 70 °C for 20 min. The mixture was diluted with aq. $NaHCO_3$ and ethyl acetate, the two phases were separated, the organic phase was dried over $MgSO_4$, filtered, and concentrated under reduced pressure. The resulting mixture was purified by column chromatography (CH_2Cl_2/MeOH/AcOH 100:20:1) to give **2** (220 mg, 17%) as an orange solid. ^{1}H NMR (300 MHz, DMSO) (*E*-isomer) δ (ppm) = 13.90 (s, 1H), 7.82 (dd, *J* = 9.2 Hz, 4H), 4.38 (q, *J* = 7.1 Hz, 2H), 1.36 (t, *J* = 7.1 Hz, 3H). HRMS-ESI: *m/z* = 369.0529 (calculated for $[M + H]^+$, 369.0498).

3: A solution of **2** (96 mg, 0.26 mM) in DMF (3 mL) together with PyBOP (162 mg, 0.31 mM), Et_3N (110 μL, 0.78 mM), and Gly-*O*-tBu·HCl (52 mg, 0.31 mM) was stirred overnight at room temperature. The mixture was then diluted with ethyl acetate and washed with brine. The two phases were separated, and the organic phase was dried over $MgSO_4$, filtered, and concentrated under reduced pressure. The resulting mixture was purified by column chromatography (ethyl acetate/hexanes 1:2) to give **3** (84 mg, 76%) as an orange solid. ^{1}H NMR (300 MHz, CD_2Cl_2) (*E*-isomer) δ (ppm) = 7.73 (d, *J* = 9.1 Hz, 2H), 7.52 (d, *J* = 9.1 Hz, 2H), 6.75 (t, *J* = 4.5 Hz, 1H), 4.38 (q, *J* = 7.1 Hz, 2H), 4.09 (d, *J* = 5.0 Hz, 2H), 1.48 (s, 9H), 1.39 (t, *J* = 7.1 Hz, 3H). HRMS-ESI: *m/z* = 482.1336 (calculated for $[M – H]^-$, 482.1339).

F-azo**:** To a solution of **3** (0.20 mM, 84 mg) in CH_2Cl_2 (5 mL) was added TFA (0.5 mL) and a drop of water. The solution turned black. After stirring 2 h at room temperature, the solution was washed with brine and evaporated under reduced pressure to give the free acid (65 mg, 0,17 mM, 88%). The free acid was then dissolved in dry THF and 60% w/w NaH/mineral oil (0.17 mM, 6 mg) was added at 0°C. The resulting mixture was allowed to come back to room temperature and stirred for 1 h. Hexane was then added, and the precipitate was filtered and dried to give F-azo (72 mg, 81%) as an orange solid. ^{1}H NMR (300 MHz, CD_2Cl_2) (*E*-isomer) δ (ppm) = 8.63 (t, *J* = 5.1 Hz, 1H), 7.82 (t, *J* = 9.6 Hz, 4H), 4.38 (q, *J* = 7.1 Hz, 2H), 3.56 (d, *J* = 5.1 Hz, 2H), 1.36 (t, *J* = 7.1 Hz, 3H). HRMS-ESI: *m/z* = 428.0880 (calculated for $[M – Na + 2H]^+$, 428.0870).

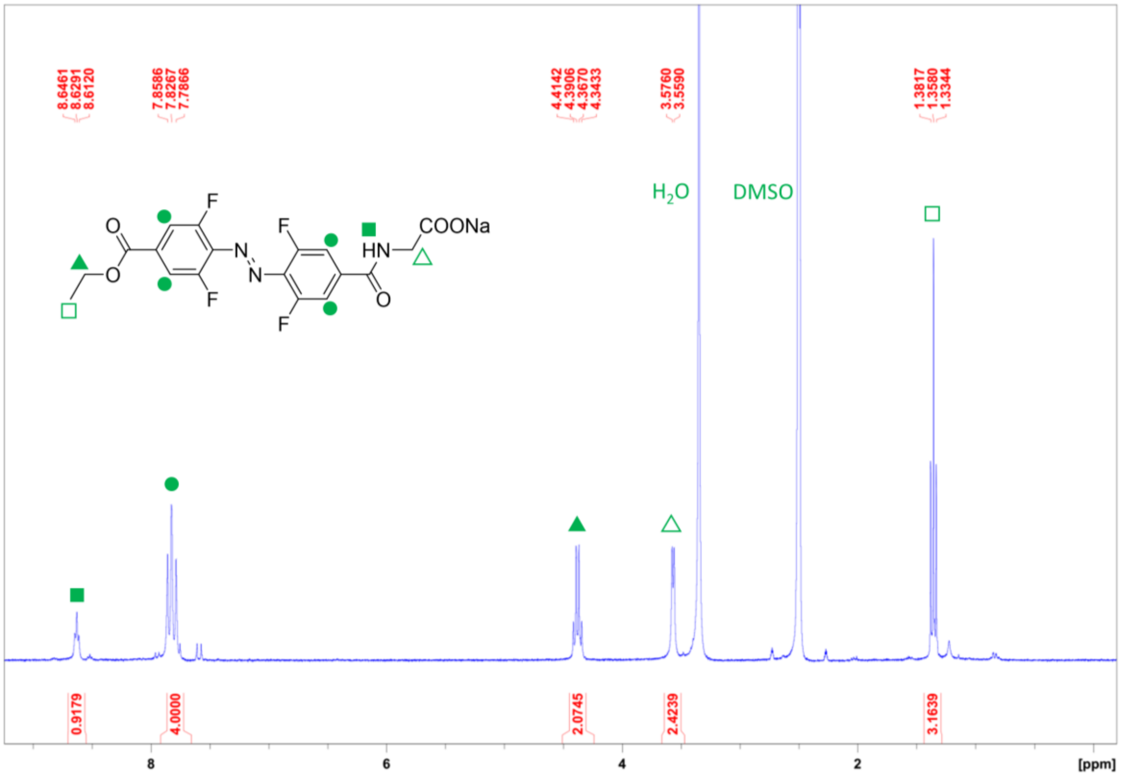

Figure S2. ^{1}H-NMR spectrum of F-azo in DMSO-*d*6.

S2. Spectral distribution of light emitted by HBO 100 mercury lamp

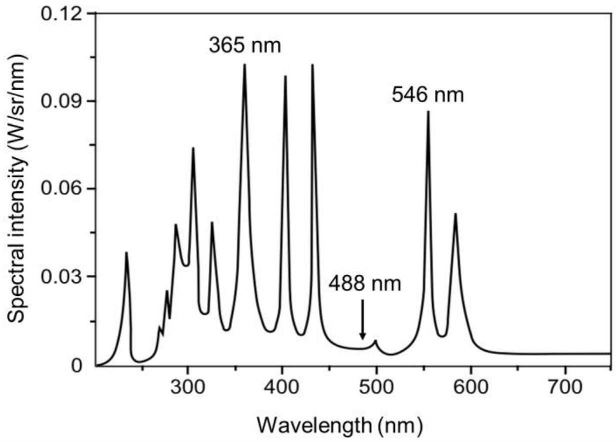

Figure S3. Spectrum of a 100 W high pressure mercury lamp. The intensity at 365 nm (the used UV range at which F-azo isomerizes to the *cis* form) is much stronger than the intensity at 488 nm (the visible range where *trans*-to-*cis* F-azo isomerization takes place). The power intensity at 546 nm measured above the objective was 0.85 mW/mm^2. It was used to estimate the power intensity at 365 nm according to the spectrum intensity.

S3. Effect of UV light (365 nm) on GUVs in the absence of F-azo.

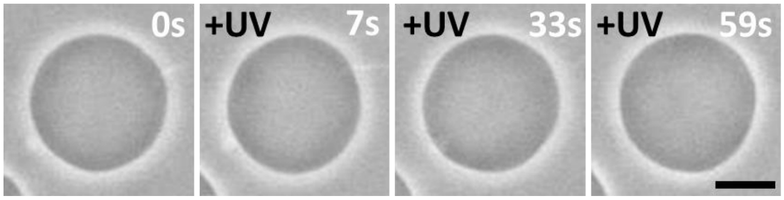

Figure S4. Time-lapse of F-azo-free GUV. A quasi-spherical vesicle was exposed to UV light in the absence of F-azo. The UV irradiation started at the 7th second. The vesicle does not undergo visible morphological changes for ~50 s of UV irradiation. The scale bar corresponds to 10 µm.

S4. Size distribution of F-azo aggregates

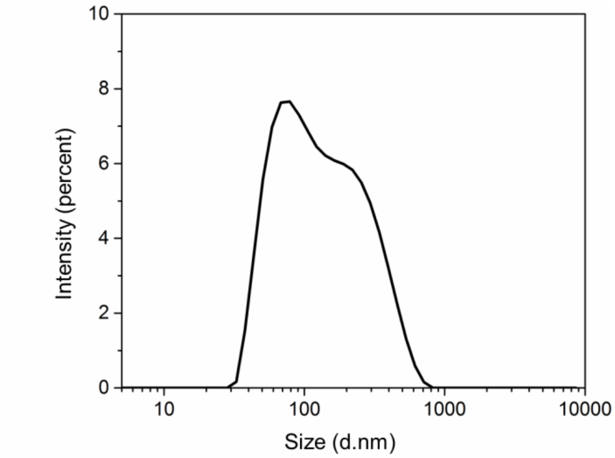

Figure S5. Size distribution of F-azo aggregates in filtered, 0.25 mM F-azo solution measured by DLS (see section 11.3 for details on measurement).

S5. Elution profile of LUVs and F-azo molecules.

The SEC system was equipped with a RF-10Axl fluorescence detector and a SPD-M10Avp diode array detector. All samples were degassed for 10 min with a degassing unit ThermoVac (MicroCal, MA) and F-azo molecules were centrifuged at 40000 rpm for 30 min at 15°C with a Sorvall Discovery M150 centrifuge (S55 rotor, Thermo Fisher Scientific, Waltham, MA, USA) before loading. 0.1 M sucrose/glucose solution was used as a solvent. Samples were loaded to the column (Superdex™ 200 10/300 GL, GE Healthcare, Freiburg, Germany) at a volume of 50 µL and runs of 60 min were performed with a flow rate of 0.3 mL/min at a maximum pressure limit of 1.1 MPa. The peaks of the F-azo elution profile in the presence of LUVs were integrated using the peak analyzer tool in OriginPro 2015. The

integration was performed with respect to an automatically calculated constant baseline, which corresponds to the minimum of the F-azo absorbance.

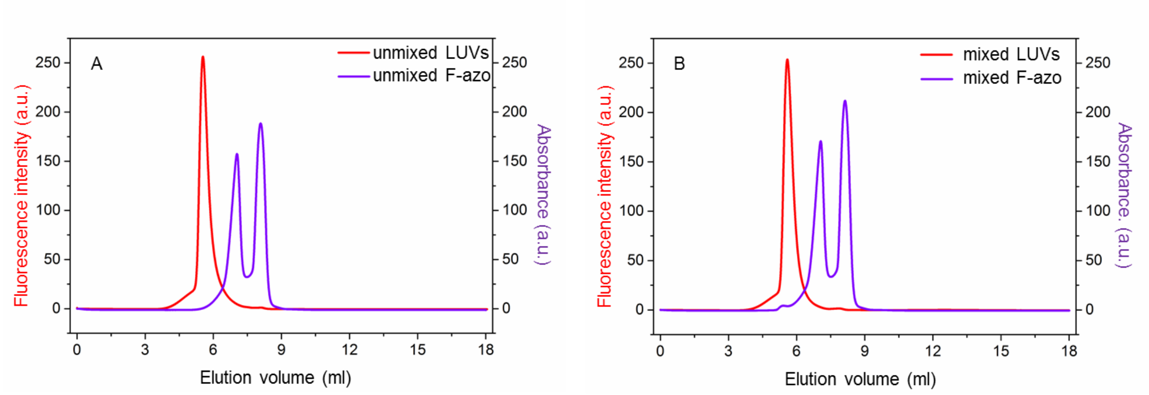

Figure S6. Chromatograms of filtered F-azo molecules (0.25 mM) and LUVs. (A) Elution profiles of unmixed solutions of LUVs and F-azo. (B) Elution of an LUV solution incubated for 2 h with F-azo. The volumes of the applied sample and the column were 50 μL and 24 mL, respectively.

S6. Electrodeformation of a GUV caused by AC field

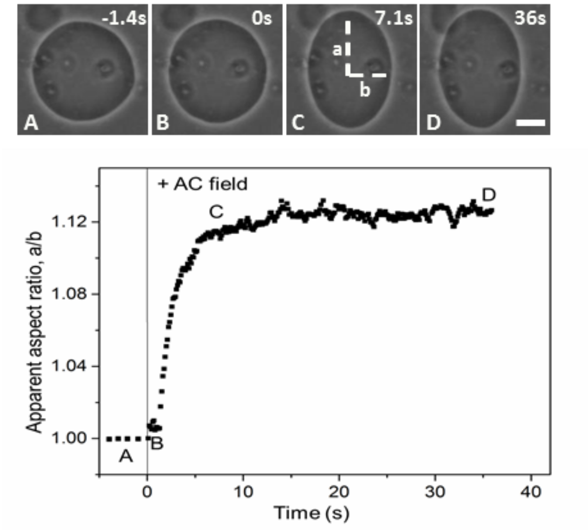

Figure. S7. Deformation of a vesicle in the presence of electric field and in the absence of F-azo. Snapshots show the dynamic of the electrodeformation. The AC field was switched on at 0 s (snapshot B) and continued for ~35 s (C and D). The graph shows the apparent aspect ratio as a function of time. The GUV reaches its maximum deformation within the first ~7 s (C). The scale bar corresponds to 10 μm.

S7. Reversible budding in the presence of an AC field.

The vesicle in Fig. S10 expels buds during UV light irradiation (snapshot B). The buds are reabsorbed by the vesicle upon blue light irradiation (snapshots C and D). The budding process repeats, when the vesicle

is irradiated with UV light for a second time (snapshot E). An AC field (1 MHz, 5 Vpp) was constantly applied. The graph shows the apparent aspect ratio of the vesicle during the process. After the budding event the vesicle deformation decreases (B and E). During the blue light irradiation, the deformation reaches its previous level due to the reabsorption of the buds. Note that the gaps in the curve, after the first UV light irradiation and before the second one, are due to the change of the filters for blue and UV light.

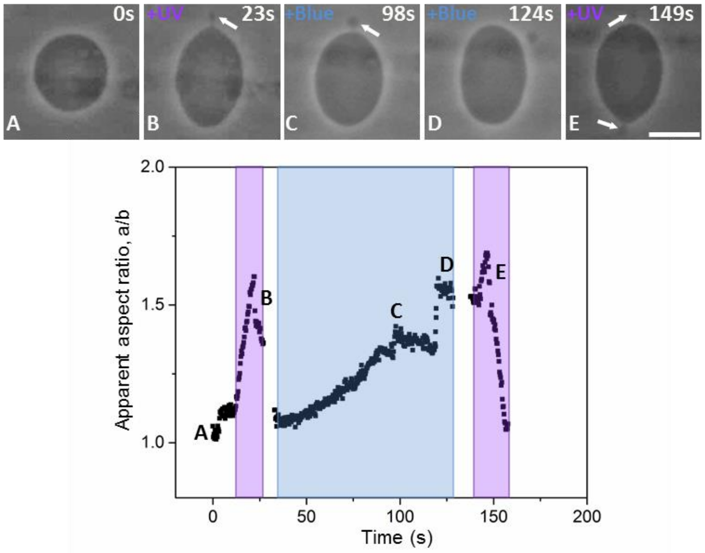

Figure S8. Degree of deformation during budding-reabsorption processes. The F-azo concentration is 0.25 mM. Snapshots show the sequence of the process. The AC field was applied at 0 s (snapshot A). The arrows in B, C and E indicate the buds. The scale bar in E corresponds to 10 µm. The graph represents the degree of the vesicle deformation. The time of UV and blue light irradiation is shown by the areas in violet and blue, respectively.

S8. MD simulations to investigate the area increase produced by the F-azo molecules

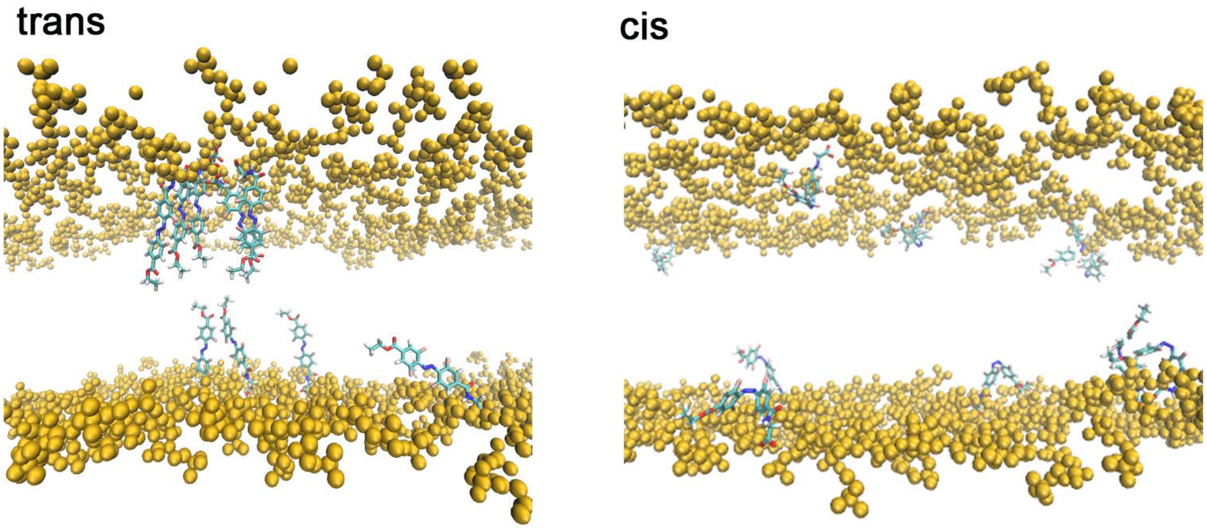

Figure S9. Simulation snapshots for bilayers with 10 trans (left) or 10 cis FAZO molecules / 256 lipids showing the clustering of *trans* F-azo.

S9. Effect of solution asymmetry on budding direction

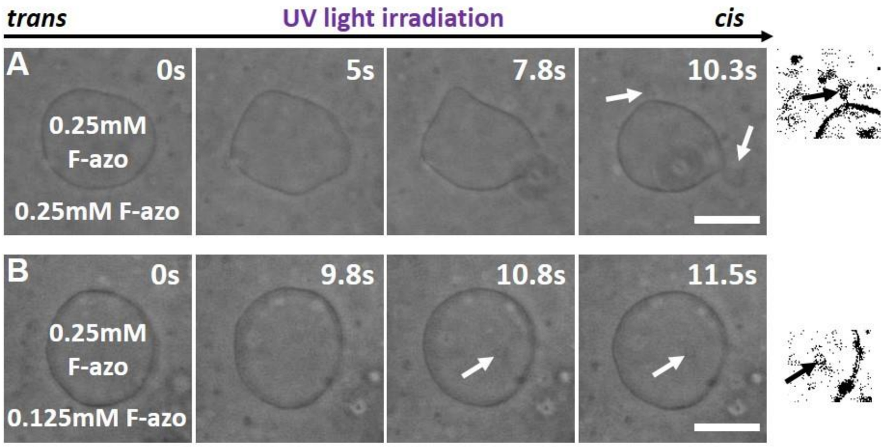

Figure S10. Time sequences of vesicle response to photoisomerization of F-azo at different dilutions of the molecules and identical glucose concentration across the membrane. The GUVs were grown in the presence of 0.25 mM F-azo and irradiated with UV light before dilution (panel A) and after twofold dilution (panels B). The vesicle in panel A exhibits outward budding, while the GUV in panels B exhibits inward budding, see cropped binary images to the right. The arrows point to the formed buds. The scale bars correspond to 20 µm.

S10. Spontaneous curvature of outward budding GUVs

The membrane area A of a vesicle defines the vesicle size

$$R_{ve} \equiv \sqrt{A/(4\pi)} \quad . \tag{1}$$

In general, for a smooth membrane surface the mean curvature is

$$M = \frac{1}{2}\,(C_1 + C_2) \tag{2}$$

where C_1 and C_2 are the principle curvatures.

Curvature elasticity according to the spontaneous curvature model:

In this model, the bending energy of any smooth shape S is given by

$$\mathcal{E}_{be}\,\{S\} = 2\kappa \int dA\,(M - m)^2 \tag{3}$$

which depends only on two parameters, the bending rigidity κ and the spontaneous curvature m. The notation $\{S\}$ is used to indicate that the bending energy represents a functional of the shape.

Curvature elasticity according to the area-difference-elasticity (ADE) model:

The ADE model is defined by the energy functional

$$\mathcal{E}_{ADE}\{S\} = \mathcal{E}_{be}\{S\} + D_{ADE}\{S\} \tag{4}$$

with the local energy term $\mathcal{E}_{be}$ defined in Eq. 3 corresponding to the spontaneous curvature model and the nonlocal area-difference-elasticity term

$$D_{ADE}\{S\} = \frac{\pi\kappa\prime}{2Al_{me}^2}\,(\Delta\mathcal{A}\{S\} - \Delta A_0)^2 = \frac{2\pi\kappa\prime}{A}(\mathcal{J}_M\{S\} - I_{M,0})^2 \tag{5}$$

where $\Delta\mathcal{A}\{S\}$ represents the area difference between the two leaflets of the vesicle with shape S,

$$\mathcal{J}_M\{S\} \equiv \int dA\,M \tag{6}$$

is the integrated mean curvature, κ' the nonlocal bending rigidity and

$$I_{M,0} = \int dA\,\frac{1}{R_{ve}} = 4\pi R_{ve} \tag{7}$$

represents the integrated mean curvature which characterizes the relaxed vesicle shape with an optimal packing of the molecules in both leaflets.

Effective spontaneous curvature:

Since the shapes that minimize the energy functional (4) of the ADE model also minimize the energy functional (3) of the spontaneous curvature model we use the effective spontaneous curvature

$$m_{eff} = m + m_{nlo} \tag{8}$$

which represents the sum of the local spontaneous curvature m with the nonlocal spontaneous curvature

$$m_{nlo} = \pi\frac{\kappa\prime}{k}\,\frac{I_{M,0} - \mathcal{J}_M\{S\}}{A} \quad . \tag{9}$$

If the relaxed vesicle state corresponds to a sphere with radius R_{ve}, one has $I_{M,0} = 4\pi R_{ve}$ and the geometric factor in (9) becomes

$$\frac{I_{M,0} - \mathcal{J}_M\{S\}}{A} = \frac{4\pi R_{ve} - \mathcal{J}_M\{S\}}{4\pi R_{ve}^2} \tag{10}$$

Neck closure condition for a spherical out-bud:

Consider a vesicle with a spherical out-bud where the mother and the bud radii are R_α and R_β, respectively. The closing neck (between the mother vesicle and the out-bud) is characterized by the condition

$$\frac{1}{R_\alpha} + \frac{1}{R_\beta} = 2m_{eff} = 2m + 2m_{nlo} \tag{11}$$

with

$$m_{nlo} = \pi \frac{\kappa'}{\kappa} \frac{R_{ve} - R_\alpha - R_\beta}{R_{ve}^2} \quad \text{and} \quad R_{ve}^2 = R_\alpha^2 + R_\beta^2 \tag{12}$$

Therefore, we can obtain the local spontaneous curvature m, if we observe the geometry of the vesicle during neck closure and use the relation

$$2m = \frac{1}{R_\alpha} + \frac{1}{R_\beta} - 2\pi \frac{\kappa'}{\kappa} \frac{\sqrt{R_\alpha^2 + R_\beta^2} - R_\alpha - R_\beta}{R_\alpha^2 + R_\beta^2} \tag{13}$$

Sphere to out-but transition curves

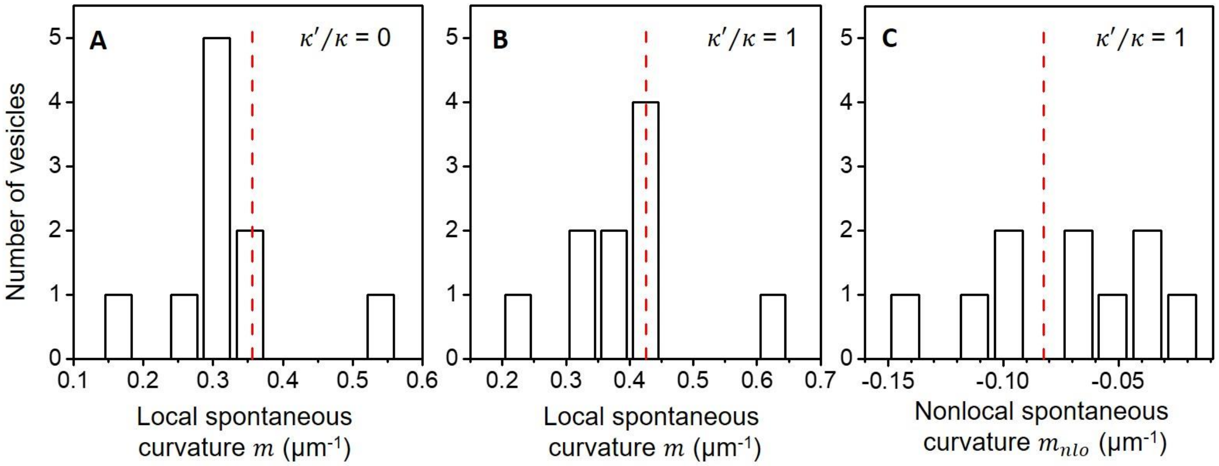

Figure S11. Histograms of the local spontaneous curvature m and the nonlocal spontaneous curvature m_{nlo}: (A) Rigidity ratio κ'/κ = 0; (B, C) Rigidity ratio κ'/κ = 1. The average values of the spontaneous curvatures are indicated by the vertical dashed lines (red).

S11. Materials and preparation of vesicles

S11.1. Materials

The lipid 1,2-dioleoyl-sn-glycero-3-phosphocholine (DOPC) and the fluorescent probe 1,2-dipalmitoyl-sn-glycero-3-phosphoethanolamine-N-(lissamine rhodamine B sulfonyl) (DPPE-Rh) were purchased from Avanti Polar Lipids (Alabaster, Al). F-azo was used as a photosensitive molecule.[1] The F-azo solutions (F-azo was dissolved in water) were stirred with a magnetic stirrer for ~ 30 min at room temperature. All

F-azo solutions used in this work were filtered through 0.22 μm filter prior to use. Sucrose and D-glucose were obtained from Sigma Aldrich (St. Louis, MO). Sodium chloride was purchased from Roth (Karlsruhe, Germany). Milli-Q water was used throughout this work.

S11.2. Vesicle preparation

Giant unilamellar vesicles were grown using the electroformation method.[2] In brief, 16 µL of a 4 mM lipid stock solution of DOPC in chloroform were spread on a pair of conductive ITO (indium tin oxide) glasses. The phospholipid film was dried under N_2 stream for 5-10 min and after that the glasses were kept under vacuum for 1-2 h to remove all traces of the organic solvent. The two glasses were placed with their conductive sides facing each other and separated by a 2 mm thick Teflon spacer. This electroswelling chamber was filled with 0.1 M sucrose solution and connected to a function generator. An AC field (1.1 V, 10 Hz) was applied for 1 h at room temperature to swell the GUVs. After the GUVs were formed they were diluted twice in an isoosmolar glucose solution containing the desired F-azo concentration. The incubation of the GUVs with F-azo lasted for 1-2 h. For the vesicle electrodeformation measurements, the GUVs were grown in the presence of 0.5 mM NaCl and 0.1 M sucrose.

Large unilamellar vesicles (LUVs) were made by extrusion. 108 µl of DOPC dissolved in chloroform (21 mM) were mixed in a glass tube with 29 µl chloroform solution of DPPE-Rh (0.8 mM). The solvent was evaporated with N_2 stream and the tube was additionally dried under vacuum for 2 h. The lipid film was then hydrated by adding a 0.1 M sucrose solution, for a final DOPC concentration of 1 mM and 1 mol% (0.01 mM) of DPPE-Rh. The obtained solution was vortexed for 5-10 min and the formed multilamellar vesicles were subjected to 20 cycles of extrusion through a polycarbonate membrane (Whatman, Maidstone, UK) with pore diameter of 100 nm. The whole procedure was performed at room temperature. Before the experiments, the LUVs were diluted 5 × in a 0.1 M sucrose solution.

S11.3. Dynamic light scattering (DLS) of F-azo samples and LUVs

The size distribution of F-azo aggregates and the extruded LUVs were determined at 25°C with a Zetasizer Nano ZS (Malvern Instruments, Worcestershire, UK), operating with a 4 mW HeNe laser (632.8 nm), a detector positioned at the scattering angle of 173°, and a temperature-control jacket for the cuvette. Aliquot of 1 ml F-azo with concentration of 0.25 mM was degassed for 10 min with ThermoVac (MicroCal, MA). Three DLS measurements consisting of 20 runs with duration of 5 s were performed. DLS measurements were performed also on degassed LUV suspensions to determine the vesicle size.

S12. F-azo aggregates

In aqueous solutions, F-azo aggregates due to its limited water solubility. Crystal-like structures were observed when using higher concentrated (> 0.5 mM) F-azo solutions (Fig. S3). In order to exclude these

crystal-like structures we filtered the F-azo stock solution (through a filter with pore size: 0.22 µm). The latter was stable and the crystal-like structures were not observed after filtration. We were concerned that the total concentration of F-azo molecules might have been reduced during the filtration step. We measured the absorbance at 320 nm of both unfiltered F-azo (in the concentration range of 0-0.1 mM) and filtered F-azo (for two concentrations: 0.025 mM and 0.05 mM). Then the extinction coefficients of the filtered and unfiltered F-azo (for 0.025 mM and 0.05 mM) were compared. The concentration of the filtered F-azo was ~ 3% lower than the one of the unfiltered F-azo solutions. This small concentration difference was considered in the calculation of the concentration of the stock F-azo solution.

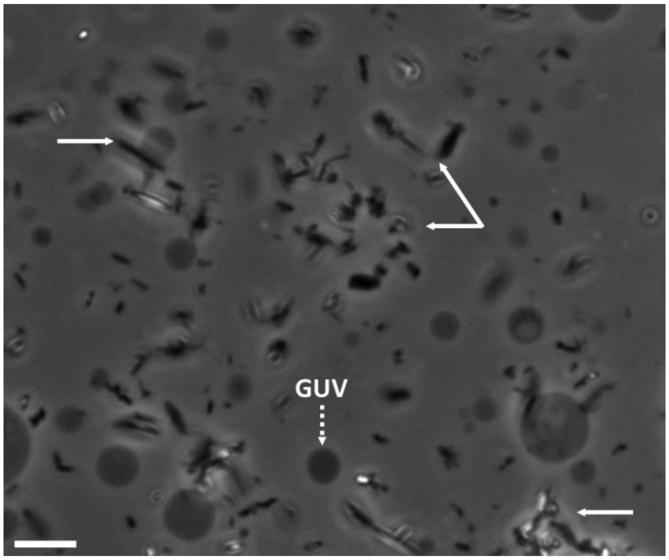

Figure S12. GUVs in the presence of unfiltered F-azo. The concentration of F-azo is 1.25 mM. The continuous arrows indicate several F-azo crystal-like aggregates. The scale bar is 10 µm.

S13. Aggregation in filtered F-azo solutions under UV light irradiation

During prolonged (~ 2 min) UV light irradiation, the F-azo molecules start form aggregates of a few µm in size (Fig. S4). Note that during our experiments, the samples were irradiated with UV light for less than a minute, which is below the threshold of the appearing of the aggregates.

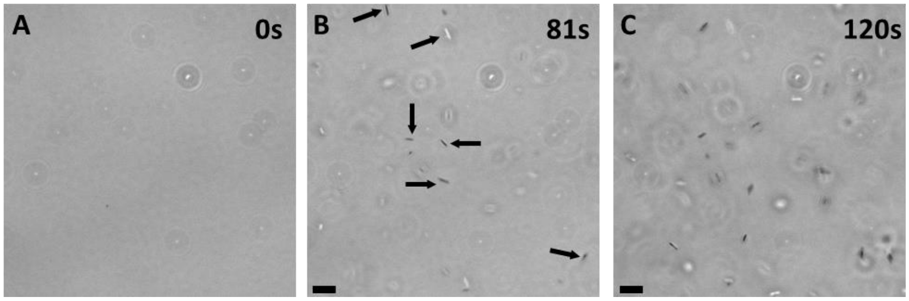

Figure S13. UV-induced aggregation of filtered F-azo (0.25 mM). The time of the irradiation is indicated on the top-right corner of each snapshot. (A) The sample before the UV light irradiation. (B) The arrows point to the first appearing aggregates. (C) More F-azo aggregates appear after further irradiation. The scale bars correspond to 10 µm.

S14. Movie S1. Reversible morphological changes in a GUV

Observation of a DOPC GUV irradiated with UV (365 nm) and blue (470 nm) light in the presence of F-azo molecules (0.25 mM). Before the observation the vesicles were dispersed in a 0.1 M glucose solution containing F-azo (0.5 mM) in volume ratio 1:1 and incubated with the photosensitive molecules for 1-2 h. The GUV changes its morphology and expels a small bud during the UV irradiation. The opposite process is observed under blue light. Picture size: 60 x 38 µm. The first part of the movie, when the vesicle is irradiated with UV light, is sped up 3 times, while the one in the presence of blue light is sped up 7 times.

S15. References

[1] D. Bleger, J. Schwarz, A.M. Brouwer, S. Hecht, *J. Am. Chem. Soc.*, 134 (2012) 20597-20600.
[2] M.I. Angelova, D.S. Dimitrov, *Faraday Discuss.*, 81 (1986) 303-311.
[3] S. Vrhovec, M. Mally, B. Kavcic, J. Derganc, *Lab Chip*, 11 (2011) 4200-4206.
